# Gender Differences in Associated and Predictive Factors of Anxiety and Depression in People With Epilepsy

**DOI:** 10.3389/fpsyt.2020.00670

**Published:** 2020-07-10

**Authors:** Zhao Liu, Rong Yin, Ze Fan, Hong Fan, Haiyan Wu, Baorui Shen, Shengxi Wu, Fang Kuang

**Affiliations:** ^1^ Department of Neurobiology, School of Basic Medicine, Fourth Military Medical University, Xi’an, China; ^2^ Department of Neurology, The 940th Hospital of Joint Logistics Support Force of People’s Liberation Army, Lanzhou, China; ^3^ Department of Anesthesiology, Xijing Hospital, Fourth Military Medical University, Xi’an, China

**Keywords:** epilepsy, anxiety, depression, psychiatric comorbidity, gender differences, risk factors

## Abstract

**Purpose:**

Comorbid anxiety and depression in people with epilepsy (PWE) are highly prevalent and contribute to low quality of life (QOL) and may even lead to poor outcomes of epilepsy. Among the various factors that affect these negative emotional comorbidities, possible gender differences remain poorly understood and are often neglected. This research aimed to determine whether there are discrepancies in the incidence and influence factors of anxiety and depression between men and women with epilepsy in a hospital in northwest China.

**Methods:**

A total of 158 adult PWE (female: N = 65; 41.1%) completed self-report questionnaires, including the Self-rating Anxiety Scale (SAS), the Self-rating Depression Scale (SDS), the Chinese version of the Quality of Life in Epilepsy-31 (QOLIE-31) inventory and the Pittsburgh Sleep Quality Inventory (PSQI). The comparison between male and female PWE was made by regression analysis.

**Results:**

For the prevalence of anxiety and depression in PWE, no gender difference was found in this study. However, the moderating factors of psychiatric comorbidities were significantly different between men and women: male PWE with comorbid anxiety were more likely to be affected by sleep quality, while anxiety symptoms in female PWE were closely associated with the frequency of seizures. Education years and QOL social function were significant indicators of depression in male PWE but not in female PWE. The important and common predictor for anxiety and depressive symptoms in PWE was QOL energy/fatigue, with male patients being more affected.

**Conclusion:**

For the PWE included in this study, the incidence of comorbid anxiety and depression in PWE was similar for men and women, but the moderating factors affecting comorbid anxiety and depressive disorders differed between genders: male PWE were more likely to be affected by psychosocial factors, while female PWE were more influenced by epilepsy itself. This exploration suggests that gender-specific health care should be considered in epilepsy therapy to improve the psychiatric condition and QOL of PWE, and different treatments should be conducted for male and female PWE to prevent negative emotional comorbidities.

## Introduction

Anxiety and depression as high-rate psychiatric comorbidities are more common in people with epilepsy (PWE) (prevalence: 20%–50%) than in the general population (prevalence: 7%–20%) (1–5)]. The relationship between epilepsy and psychiatric disturbance is bidirectional; specifically, PWE are more prone to develop psychiatric comorbidities, while patients with certain primary psychiatric disorders are at higher risk of developing epilepsy (6). This bidirectional relation may be explained by the common pathogenesis of both epilepsy and psychiatric disorders (7). Psychiatric comorbidities, especially depression and anxiety, impact seizure disorders and the lives of PWE. To improve quality of life and promote efficiency in the treatment of epilepsy, screening for anxiety and depression and seeking for possible causes should become a necessary clinical routine. Unfortunately, psychiatric comorbidities often go unrecognized and untreated in PWE (8).

Although studies have found that women usually experience higher levels of anxiety and depression than men in the general population (9, 10), little is known about the gender differences in comorbid psychological disorders in epilepsy. So far, only one study has estimated the gender differences in the incidence of comorbid anxiety and depression in PWE and found that female patients with epilepsy are more susceptible to be affected by depression than male patients, but there is no gender difference in the prevalence of anxiety [(10). Therefore, more evidence is needed to increase the understandings of the gender differences in comorbid anxiety and depression in PWE.

Based on the gender discrepancy in psychiatric disorders, we hypothesized that there should be differences between male and female PWE in comorbid anxiety and depression. So the objective of the present study was to seek out gender differences in sociodemographic, clinical, incidence and risk factors of epilepsy comorbid anxiety/depression symptoms by investigating adult PWE in our hospital located in northwest China. Using statistical analyses of data obtained by self-rating questionnaires, we found differences between genders in the most predictors but not prevalence of epilepsy comorbid negative emotions.

## Subjects and Methods

### Participants and Recruitment

All 158 (male 93, female 65) adult PWE were recruited from the outpatient and inpatient Department of Clinical Neurology, the 940th Hospital of Joint Logistics Support Force of People’s Liberation Army (Lanzhou, Gansu Province, China). Patients were consecutively enrolled in this study between March 2017 and November 2017. The included patients should meet following criteria: (1) older than 18 years, (2) no other systemic disease, and (3) epilepsy was diagnosed by experienced epileptologists according to the International League against Epilepsy, (4) cognitively capable of communicating with physicians and understanding the questionnaires. After recruited the patients, resident doctors sent the questionnaires to the patients and helped them to fill in; physicians, attending doctors were responsible for collecting and analyzing data.

This study was approved by the Institutional Review Board prior to initiation, and each participant was consent and signed the patient’s medical notes to collect demographic and clinical information, including age, gender, years of education, occupational status, age at onset, duration of seizures, the number of antiepileptic drugs (AEDs) used daily, frequency of AED use, self-reported adverse effects of AEDs and frequency of seizures.

### Assessment Scales

#### Self-Rating Anxiety Scale and Self-Rating Depression Scale

We adopted the Self-rating Anxiety Scale (SAS) and the Self-rating Depression Scale (SDS) to assess anxiety and depression in the enrolled PWE. The SAS and SDS are widely used questionnaires among PWE, and their Chinese versions were administered in a previous study (11, 12). Either the SAS or the SDS composes 20-item symptom inventories, and each item is rated on a scale from 1 to 4. The total score is multiplied by 1.25 and is then converted into a standardized score ranging from 25 to 100, with higher scores reflecting more severe anxiety and depression. The criteria of severities applied in China are as the following: mild anxiety (score 50–60) and mild depression (score 53–62), moderate anxiety (score 61–70) and moderate depression (score 63–72), and severe anxiety (score>70) and severe depression (score>72) (13).

#### Quality of Life in Epilepsy-31 Inventory

Quality of life was assessed using the Chinese version of the Quality of Life in Epilepsy-31 (QOLIE-31) inventory, which is widely acknowledged epilepsy-specific QOL instruments and shows good validity and reliability in Chinese populations (14–16). The QOLIE-31 contains seven multi-item scales: seizure worry, overall quality of life (QOL), emotional well-being, energy/fatigue, cognitive function, medication effects and social function. The QOLIE-31 overall score is the sum of the weighted average of each subscale score, ranging from 1 to 100, with higher scores meaning a more favorable QOL (17, 18).

#### Pittsburgh Sleep Quality Inventory

The Pittsburgh Sleep Quality Inventory (PSQI) is a self-report questionnaire for assessing sleep. The global score ranges from 0 to 21, with a higher score indicating poorer sleep quality. The scale consists of seven subscales, comprising subjective sleep quality, sleep latency, sleep duration, sleep efficiency, sleep disturbance, use of sleep medications and daytime dysfunction. A total PSQI score of 5 or more points indicates poor sleep quality, and more than 10 points is considered severely disturbed sleep (19).

### Statistical Analysis

All data were analyzed using the software package SPSS 23.0. Descriptive statistics were calculated for all variables. Quantitative data are expressed as the mean ± standard deviation (SD). Qualitative data are shown and summarized as numbers and proportions.

Gender differences were compared using Student’s t-test for continuous variables and Chi-square tests for categorical variables. To explore the relationships between depression/anxiety and sociodemographic, psychosocial and clinical variables, Pearson’s r was calculated. The independent variables correlating with anxiety or depression, which were defined as the scores of SAS (≥ 50) and SDS (≥ 53), respectively, were introduced into a logistic regression model with stepwise selection. All tests for statistical significance were two-sided, and *p* < 0.05 was considered significant.

## Results

### Demographic and Clinical Characteristics

**Figure 1:**
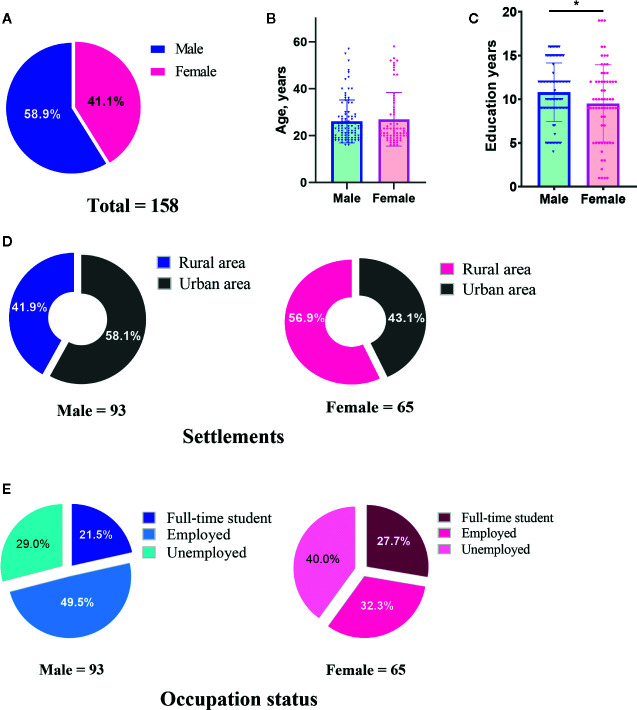
Demographic characteristics of male and female patients with epilepsy included in this study. The number of cases **(A)**, ages **(B)**, education years **(C)**, settlements **(D)** and occupation status **(E)** of male and female patients were indicated, respectively.

**Figure 2:**
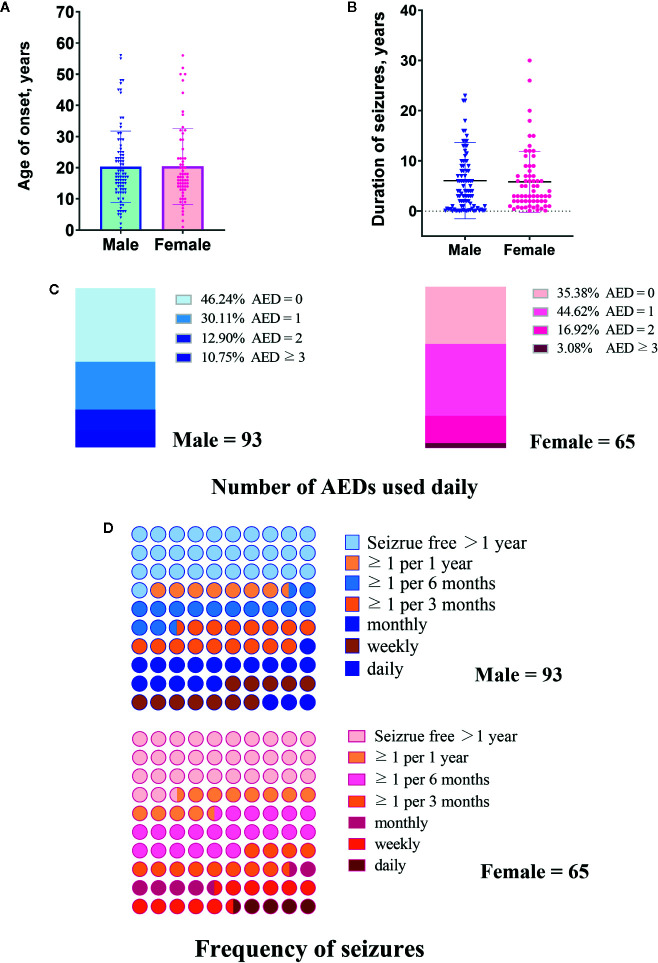
Clinical characteristics of male and female patients included in this study. **(A)** Age of onset years. **(B)** Duration of seizures. **(C)** Number of antiepileptic drugs (AEDs) used daily. **(D)** Frequency of seizures.

The demographic and clinical characteristics of 158 PWE (93 male and 65 female) are summarized in **Table 1** and **Figures 1** and **2**. Most parameters were similar in male and female patients. The significant difference between genders was only found in terms of education years in which male patients had a longer education time than female patients (**Figure 1C**).

**Table 1:** Gender differences in demographic and clinical characteristics.

Characteristics	Total sample(N = 158)	Men(N = 93)	Women(N = 65)	*p*
Gender, n (%)				
Male	93 (58.9)	93 (100)	–	
Female	65 (41.1)	–	65(100)	
Age, years (mean ± SD)	26.39 ± 10.10	26.02 ± 9.11	26.92 ± 11.44	0.583
Settlement, n (%)				0.064
Rural area	76 (48.1)	39 (41.9)	37 (56.9)	
Urban area	82 (51.9)	54 (58.1)	28 (43.1)	
Education years (mean ± SD)	10.27 ± 3.86	10.80 ± 3.35	9.52 ± 4.42	0.041
Occupation status, n (%)				0.098
Full-time student	38 (24.1)	20 (21.5)	18 (27.7)	
Employed	67 (42.4)	46 (49.5)	21 (32.3)	
Unemployed	53 (33.5)	27 (29.0)	26 (40.0)	
Age of onset, years (mean ± SD)	20.33 ± 3.86	20.28 ± 11.47	20.41 ± 12.22	0.946
Duration of seizures, years (mean ± SD)	5.98 ± 7.00	6.07 ± 7.59	5.83 ± 6.08	0.833
Number of AEDs used daily, n (%)				0.082
0	66 (41.8)	43 (46.2)	23 (35.4)	
1	57 (36.1)	28 (30.1)	29 (44.6)	
2	23 (14.6)	12 (12.9)	11 (16.9)	
≥3	12 (7.6)	10 (10.8)	2 (3.1)	
Frequency of seizures, n (%)				0.319
Seizrue free > 1 year	50 (31.7)	29 (31.2)	21 (32.3)	
≥1 per 1 year	15 (9.5)	7 (7.5)	8 (12.3)	
≥1 per 6 months	27 (17.1)	13 (14.0)	14 (21.5)	
≥1 per 3 months	23 (14.6)	15 (16.1)	8 (12.3)	
monthly	19 (12.0)	15 (16.1)	4 (6.2)	
weekly	18 (11.4)	11 (11.8)	7 (10.8)	
daily	6 (3.8)	3 (3.2)	3 (4.6)	
Type of epilepsy, n (%)				0.662
Generalized epilepsy	138 (87.3)	82 (88.2)	56 (86.2)	
Partial epilepsy	17 (10.8)	10 (10.8)	7 (10.8)	
Unclassified epilepsy	3 (1.9)	1 (1.1)	2 (3.1)	

AEDs, antiepileptic drugs; SD, standard deviation.

### Prevalence of Anxiety and Depressive Symptoms

**Figure 3:**
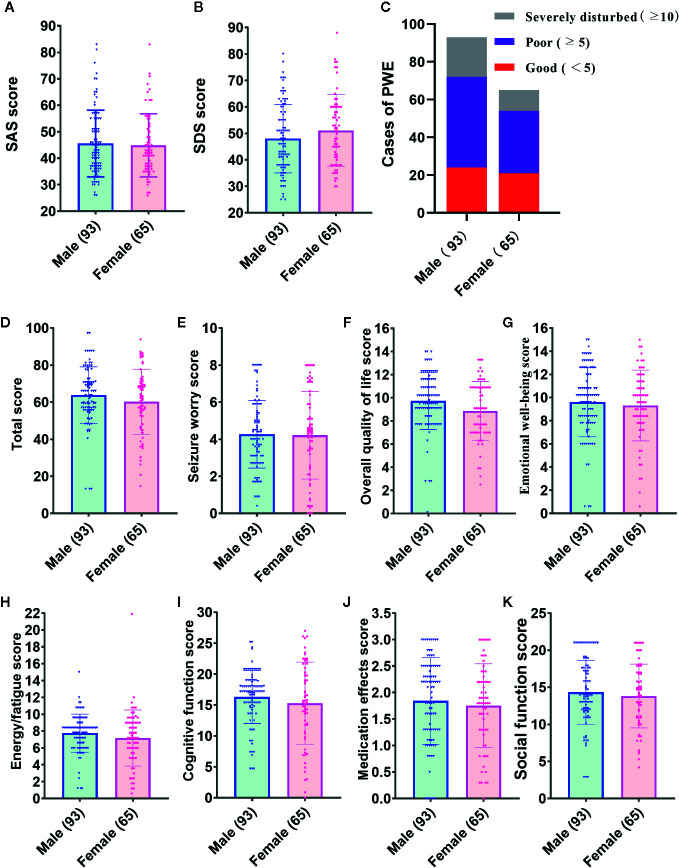
Inventory scores of male and female patients included in this study. **(A)** Self-rating Anxiety Scale (SAS) score. **(B)** SAS score. **(C)** Cases of people with epilepsy (PWE) in different grades of sleep quality. **(D–K)** Total and subscale scores of the Quality of Life in Epilepsy-31 (QOLIE-31) of seizures.

The SAS and SDS scores for all participants are listed in **Table 2** and **Figures 3A, B**. Of the 158 patients, the mean overall scores of the SAS and SDS were 45.28 (SD = 12.29) and 49.28 (SD = 13.20), respectively. No significant difference was found between genders in the percentage of subjects with high SAS and SDS scores.

**Table 2:** Mood self-evaluation inventory scores.

Items	Total sample(N = 158)	Men(N = 93)	Women(N = 65)	*p*
Anxiety and depression				
SAS (mean ± SD)	45.28 ± 12.29	45.56 ± 12.59	44.88 ± 11.92	0.732
Normal, n (%)	108 (68.4)	61 (65.6)	47 (72.3)	0.671
Mild	28 (17.7)	19 (20.4)	9 (13.8)	
Moderate	13 (8.2)	7 (7.5)	6 (9.2)	
Severe	9 (5.7)	6 (6.5)	3 (4.6)	
SDS (mean ± SD)	49.28 ± 13.20	48.75 ± 13.62	50.03 ± 12.65	0.551
Normal, n (%)	100 (63.3)	57 (51.6)	43 (66.2)	0.773
Mild	31 (19.6)	20 (21.5)	11 (16.9)	
Moderate	17 (10.8)	11 (20.4)	6 (9.2)	
Severe	10 (6.3)	5 (6.5)	5 (7.7)	

### Other Inventory Results

The overall and subscale scores of the QOLIE-31 and PSQI are shown in **Table 3** and **Figure 3**. For the overall sample, the mean overall QOLIE-31 score was 62.29 (SD = 16.30). According to the PSQI rating scale, only 28.5% of the total PWE participated declared good quality of sleep, but 51.3% had poor sleep, and 20.3% reported severely disturbed sleep, respectively (**Figure 3C**). The female PWE had higher Sleep Quality score than the male (*p* = 0.023), and no significant gender difference was found in terms of any other total or subscale scores of the PSQI and QOLIE-31 (**Figures 3D–K**).

**Table 3:** Other inventory scores.

Items	Total sample **(N = 158)**	Men **(N = 93)**	Women **(N = 65)**	*p*
QOLIE-31				
Total score (mean ± SD)	62.29 ± 16.30	61.48 ± 17.03	63.45 ± 15.25	0.467
Seizure worry	4.24 ± 2.05	4.42 ± 2.13	4.28 ± 1.95	0.848
Overall quality of life	9.37 ± 2.52	9.11 ± 2.75	9.74 ± 2.11	0.124
Emotional well-being	9.48 ± 3.00	9.52 ± 3.12	9.42 ± 2.86	0.841
Energy/fatigue	7.51 ± 2.76	7.36 ± 2.65	7.72 ± 2.91	0.421
Cognitive function	15.88 ± 5.38	15.84 ± 5.69	15.95 ± 4.93	0.896
Medication effects	1.81 ± 0.81	1.84 ± 0.80	1.75 ± 0.81	0.493
Social function	14.12 ± 4.30	13.76 ± 4.52	14.62 ± 3.96	0.217
PSQI, n (%)				
Good (<5)	45(28.5)	24 (25.8)	21 (32.3)	0.555
Poor (≥5)	81(51.3)	48 (51.6)	33 (50.8)	
Severely disturbed (≥10)	32(20.3)	21 (22.6)	11 (16.9)	
Total score (mean ± SD)	6.75 ± 3.97	7.12 ± 4.10	6.23 ± 3.75	0.168
Sleep quality	1.08 ± 0.78	1.19 ± 0.81	0.91 ± 0.70	0.023
Sleep latency	1.32 ± 1.01	1.37 ± 1.00	1.25 ± 1.02	0.481
Sleep duration	0.66 ± 0.83	0.70 ± 0.87	0.62 ± 0.76	0.533
Sleep efficiency	0.76 ± 0.96	0.81 ± 0.98	0.70 ± 0.94	0.510
Sleep disturbances	1.27 ± 0.85	1.32 ± 0.92	1.20 ± 0.74	0.421
Sleep medication	0.11 ± 0.54	0.10 ± 0.53	0.12 ± 0.55	0.763
Daytime dysfunction	1.58 ± 1.17	1.66 ± 1.26	1.48 ± 1.03	0.347

### Correlation Analysis

#### Gender Differences in the Correlation Between Clinical Variables and Anxiety (SAS Scores)

**Table 4** outlines a univariate correlation analysis between various factors and SAS scores in men and women with epilepsy. The correlation analysis revealed that anxiety (SAS scores ≥50) in both men and women with epilepsy was positively correlated with the frequency of seizures (men: r = 0.207, *p* < 0.05; women: r = 0.467, *p* < 0.05), the overall QOLIE-31 scores (men: r = −0.522, *p* < 0.01; women: r = -0.606, *p* < 0.01) and the PSQI scores (men: r = 0.467, *p* < 0.01; women: r = 0.445, *p* < 0.01). In addition, anxiety in women with epilepsy was positively correlated with occupation (r = −0.394, *p* < 0.01), years of education (r = −0.422, *p* < 0.01) and settlement (r = −0.330, *p* < 0.01). There were also significant differences in the correlations between anxiety and each subscale domain of the QOLIE-31 and PSQI, which are listed in **Table 4**.

**Table 4:** Correlation analysis of factors influencing mood of patients with epilepsy.

Variables	Anxiety (SAS)		Depression (SDS)	
	Men(N=93)	Women(N=65)	Men(N=93)	Women(N=65)
Demographic characteristics				
Age	−0.007	0.156	−0.073	0.152
Occupation	0.102	−0.394^**^	0.125	−0.400^**^
Education years	−0.153	−0.422^**^	−0.292^**^	−0.416^**^
Settlement	−0.027	−0.330^**^	0.004	−0.360^**^
Clinical characteristics				
Onset age	−0.088	0.010	−0.081	−0.033
Duration	0.175	0.081	0.026	0.111
Numberof AEDs	0.151	−0.075	0.090	0.064
Frequency of seizures	0.207^*^	0.467^**^	0.230^*^	0.377^**^
Type of epilepsy	0.072	−0.248^*^	0.096	−0.205
QOLIE-31				
Total score	−0.522^**^	−0.606^**^	−0.564^**^	−0.557^**^
Seizure worry	−0.248^*^	−0.579^**^	−0.303^**^	−0.544^**^
Overall quality of life	−0.371^**^	−0.251^*^	−0.429^**^	−0.262^*^
Emotional well-being	−0.521^**^	−0.576^**^	−0.511^**^	−0.642^**^
Energy/fatigue	−0.654^**^	−0.697^**^	−0.675^**^	−0.676^**^
Cognitive function	−0.432^**^	−0.392^**^	−0.423^**^	−0.363^**^
Medication effects	−0.225^*^	−0.338^**^	−0.181	−0.260^*^
Social function	−0.306^**^	−0.416^**^	−0.441^**^	−0.348^**^
PSQI				
Total score	0.467^**^	0.445^**^	0.388^**^	0.330^**^
Sleep quality	0.318^**^	0.277^*^	0.263^*^	0.287^*^
Sleep latency	0.362^**^	0.370^**^	0.280^**^	0.324^**^
Sleep duration	0.145	0.109	0.113	0.015
Sleep efficiency	0.117	0.182	0.079	0.162
Sleep disturbances	0.468^**^	0.342^**^	0.348^**^	0.291^*^
Sleep medication	0.252^*^	0.275^*^	0.230^*^	0.227
Daytime dysfunction	0.402^**^	0.483^**^	0.344^**^	0.306^*^

^*^ p ＜ 0.05.

^**^ p ＜ 0.01.

#### Gender Differences in the Correlation Between Clinical Variables and Depression (SDS Score)

The correlational analysis showed that depression in men with epilepsy was positively correlated with education years (r = −0.279, *p* < 0.01), frequency of seizures (r = 0.321, *p* < 0.01), QOLIE-31 scores (r = −0.615, *p* < 0.01) and PSQI scores (r = −0.501, *p* < 0.01). In contrast, depression in women with epilepsy was positively correlated with occupation (r = −0.394, *p* < 0.01), years of education (r = −0.422, *p* < 0.01), settlement (r = −0.330, *p* < 0.01), QOLIE-31 scores (r = −0.615, *p* < 0.01) and PSQI scores (r = −0.501, *p* < 0.01).

### Logistic Regression Analysis

#### Gender Differences in Predictors of Anxiety

Logistic regression models for the male group explained the independent predictors of the SAS score. These predictors included energy/fatigue (OR = 0.432) as the subscale of the QOLIE-31 and the PSQI score (OR = 1.339) (**Table 5**). In the female group, energy/fatigue (OR = 0.151) and frequency of seizures (OR = 3.001) significantly predicted the SAS score (**Table 5**).

**Table 5:** Logistics analysis for anxiety in men or women with epilepsy.

	B	S.E.	Wald	OR	*p*
**Men**					
Duration	0.101	0.058	3.095	1.106	0.079
Energy/fatigue (QOLIE-31)	−0.839	0.217	14.991	0.432	<0.001
PSQI	0.292	0.107	7.491	1.339	0.006
**Women**					
Frequency of seizures	1.099	0.517	4.517	3.001	0.034
Seizure worry (QOLIE-31)	−0.873	0.586	2.217	0.418	0.137
Energy/fatigue (QOLIE-31)	−1.891	0.728	6.751	0.151	0.009

#### Gender Differences in Predictors of Depression

Logistic regression analysis indicated that education years (OR = 0.769), energy/fatigue (OR = 0.482) and social function (OR = 0.698), as the subscales of the QOLIE-31, independently predicted depression for the male group (**Table 6**). In the female group, only energy/fatigue (OR = 0.265) was a significant indicator of depression (**Table 6**).

**Table 6:** Logistics analysis for depression in men or women with epilepsy.

	B	S.E.	Wald	OR	*p*
**Men**					
Education years	−0.263	0.119	4.873	0.769	0.027
Energy/fatigue (QOLIE-31)	−0.729	0.194	14.163	0.482	0.000
Medication effects (QOLIE-31)	0.907	0.498	3.314	2.477	0.069
Social function (QOLIE-31)	−0.360	0.124	8.426	0.698	0.004
PSQI	0.163	0.097	2.863	1.177	0.091
**Women**					
Seizure worry (QOLIE-31)	−0.621	0.348	3.186	0.537	0.074
Energy/fatigue (QOLIE-31)	−1.328	0.398	11.108	0.265	0.001
Medication effects (QOLIE-31)	1.762	0.757	5.417	5.826	0.202
MoCA	−0.201	0.104	3.736	0.818	0.053

## Discussion

Gender discrepancy usually exists in psychiatric disorders. However, that difference(s) between genders may not always be the same in all kinds of scenarios. The current study investigated gender differences in the demographic variables, incidence and risk factors of comorbid anxiety and depression in PWE and have three key findings in the present study: (1) Expected gender differences in the prevalence of anxiety and depression are not actually seen in PWE. (2) Most of the important predictors for anxiety and depression in PWE vary by gender. (3) QOL energy/fatigue is the common influential factor of anxiety and depression in PWE and influences male and female PWE to different extents.

Large-scale epidemiological studies suggest that anxiety disorders are much more prevalent in women than in men, and this gender disparity is even more pronounced in other neuropsychiatric disorders, such as sleep disturbance (20). However, this study revealed no gender difference in the high prevalence rates of comorbid anxiety in PWE, which was consistent with previous studies (21, 22). Women in general are also documented to be more prone to depression than men (9, 10, 23), and recent evidence (22) also showed the female PWE had a higher ratio of comorbid depressive symptoms than the male. Paradoxically, the present study showed that men with epilepsy were just as likely as female patients to exhibit markedly higher levels of depression in comparison to the general population. Such discrepancies may be attributed to the different features of the selected samples among these studies. Significantly, the patients in this study were mainly from Gansu, a province of northwest China where economy and healthcare conditions are relatively under-developed. The unemployed and poorly educated situations may greatly impact the emotions of those participants. Nevertheless, our findings indicate that psychiatric comorbidities in male PWE must be concerned as important as in female patients, and the psychological state of PWE should not be equated with that of the general population.

Although the expected gender difference in the prevalence of anxiety and depression in PWE was not observed in this study, the important predictors of comorbid anxiety were found in this study quite complex and differed between male and female PWE. In men with epilepsy, poor sleep is the most dangerous factor in comorbid anxiety disorders. Sleep problems occur more frequently in PWE than in healthy controls (24). Sleep disturbance is a well-documented risk factor for developing or worsening anxiety disorders (25, 26), and women in the general population are more likely than men to report insomnia (25–27). Surprisingly, this study showed that men and women with epilepsy suffered equally from sleep problems. In contrast, the frequency of seizures was the most important indicator that independently related to anxiety in women with epilepsy in this study. Anxiety and depression are always correlated with seizure frequency, both before and after treatment, and depressive symptoms and seizure frequency influence mutually, indicated by both cross-sectional and longitudinal studies (28, 29). Our data are consistent with this finding in terms of anxiety but not depression. The analysis showed that anxiety symptoms associated seizure frequency stronger in women than in men in this study. This phenomenon may result from women’s higher level of psychological burden when facing a higher frequency of seizures, compared with male patients. Therefore, seizure-related factors impact the anxious status of women much more in comparison with men. For female PWE, good control of epilepsy and maintaining a positive and optimistic attitude toward seizures may help to reduce anxiety.

Predictors of depressive symptoms in PWE also varied by gender. It was notable in this study that depression was independently associated with education years and QOL social function in men with epilepsy but not in women. As reported, epilepsy often leads to poorer educational and vocational outcomes including unemployment and limited career prospects, especially for male patients (30). Data in the present study indicated that longer education years and better social function meant a lower risk of depression; on the contrary, poorly educated male PWE may have more psychological barriers and less-flexible coping strategies. In general, social support means differently to men and women. Women commonly relied on searching for social and religious support, while most men showed alexithymia and more difficulties on coping negative emotions (31), which may explain the gender differences in the predictors of depression in PWE in our study.

Our findings also suggest the importance of the relationship between fatigue and mental comorbidity in people with epilepsy. QOL medication effects, as the most important indicators of depression in both genders (32), were significant in our regression model of men with epilepsy. In women with epilepsy, rather than sociodemographic factors, QOL energy/fatigue almost exclusively account for the variance in depression. The degree of fatigue was found higher in adult PWE than in the general population (4, 33). In this study, the bidirectional relationship between negative emotions and epilepsy was well established: QOL energy/fatigue was confirmed as the common predictor of comorbid anxiety and depression in both genders with epilepsy. Moreover, the male patients were influenced by QOL energy/fatigue to a greater extent than the female patients. Although the mechanism of fatigue remains unclear, previous studies have found that fatigue was prominent in people with major depressive disorder even when depression was atypical (34). Thus, the association between depression (or anxiety) and fatigue should be fully understood, and fatigue should particularly be ameliorated in epilepsy patients. Seizure frequency and sleep-related problems have been found crucial risk factors for fatigue in epilepsy patients (33, 35), which may help to explain why PWE with anxiety disorders share fatigue as a common risk factor without gender differences.

In clinical practice, the main reason for psychiatric disorders in PWE usually is orchestration of large number of factors. However, our findings uncovered some interesting correlations between those factors, most of which serving as predictors for depression seem regulatable. Our study suggests that male and female PWE should be treated differently, particularly regard to negative emotional comorbidities. Further social support for male PWE and effective health education on epilepsy for female PWE may increase their confidence in treatment and relieve their anxiety about seizures.

### Limitations

The following limitations should be considered when our data are being interpreted. (1) The cases were acquired from a single epilepsy center, and the sample size was relatively small. Thus the conclusion from these data cannot be applied to other patient groups directly. (2) A definitive diagnosis of depression or anxiety disorders should meet the Diagnostic and Statistical Manual of Mental Disorders (DSM) criteria by a comprehensive clinical examination. The SAS and SDS are brief screening instruments to assess the severity of anxiety or depressive symptoms and more appropriate for epidemiological research. (3) The data in our study were collected by participants’ self-reports with no confirmation by professional psychiatric staff. Therefore, the patients may have over-evaluated or underreported their actual situation of epilepsy. (4) The main type of epilepsy in most PWE of our sample is generalized epilepsy, while focal epilepsy has a higher prevalence in the adults.

## Conclusion

The data of this study suggest that there may be no gender difference in the prevalence of epilepsy comorbidity of anxiety and depression, but gender discrepancy plays a significant role in the incidence of psychiatric comorbidities in epilepsy. Most of the important predictors for anxiety and depression in PWE vary between genders: male patients are more likely to be affected by psychosocial factors, while female patients are more influenced by seizure-related events. In addition, common indicators (e.g., energy/fatigue) may influence the psychiatric comorbidities of epilepsy differently in male and female PWE. To improve the psychiatric condition and QOL of PWE, psychological interventions and related medical care should be tailored according to gender-specific predictors.

## Data Availability Statement

The raw data supporting the conclusions of this article will be made available by the authors, without undue reservation.

## Ethics Statement

The studies involving human participants were reviewed and approved by The Ethics Committee of the 940th Hospital of Joint Logistics Support force of committee: Chinese People’s Liberation Army (Department of Neurology, The 940th Hospital of Joint Logistics Support Force of People’s Liberation Army, Lanzhou, Gansu, China.). The patients/participants provided their written informed consent to participate in this study.

## Author Contributions

SW and FK conceived of and designed the study. ZL, RY, ZF, HF, HW, and BS were involved in data acquisition. ZL and FK analyzed the data and wrote the manuscript. All authors contributed to the article and approved the submitted version.

## Funding

This work was funded by grants to SW (Innovation Teams in Priority Areas Accredited by the Ministry of Science and Technology, No. 2014RA4029) and ZL (Scientific Research Project of health industry in Gansu Province, No. GSWSKY-2019-64).

## Conflict of Interest

The authors declare that the research was conducted in the absence of any commercial or financial relationships that could be construed as a potential conflict of interest.
